# Anti-NXP2 antibody-positive dermatomyositis presenting as systemic capillary leak syndrome with cutaneous and gastrointestinal ulcers

**DOI:** 10.1093/rap/rkag022

**Published:** 2026-02-13

**Authors:** Rikako Nakamura, Yusuke Sugimori, Jun Makino, Hiroya Kuwahara, Naoko Okiyama, Takuji Nishikawa, Kenichi Shimane

**Affiliations:** Department of Rheumatology, Tokyo Metropolitan Bokutoh Hospital, Tokyo, Japan; Department of Rheumatology, Tokyo Metropolitan Bokutoh Hospital, Tokyo, Japan; Department of Critical Care Medicine, Tokyo Metropolitan Bokutoh Hospital, Tokyo, Japan; Department of Neurology and Neurological Science, Graduate School of Medical and Dental Sciences, Institute of Science Tokyo, Tokyo, Japan; Department of Dermatology, Graduate School of Medical and Dental Sciences, Institute of Science Tokyo, Tokyo, Japan; Department of Rheumatology, Tokyo Metropolitan Bokutoh Hospital, Tokyo, Japan; Department of Rheumatology, Tokyo Metropolitan Bokutoh Hospital, Tokyo, Japan

Key message

- Anti-NXP2 antibody-positive dermatomyositis involves severe vasculopathy-related complications such as systemic capillary leak syndrome.

Dear Editor, Anti-nuclear matrix protein 2 (NXP2) antibody is a major myositis-specific antibody in JDM, accounting for ≈20% of cases [1]. Adult cases with anti-NXP2 antibody often lack the characteristic rash and are frequently associated with malignant tumours [2]. Here we report a patient with adult-onset anti-NXP2 antibody-positive dermatomyositis complicated by vasculopathy such as systemic capillary leak syndrome (SCLS).

A 44-year-old man with hypertension and obesity presented with painful oral ulcers, muscle pain in both thighs, dysphagia, shortness of breath, facial erythema and low-grade fever 16 days prior to his visit. One day prior to his visit he was noted at another hospital to have hypotension. Blood tests revealed elevated creatine kinase (CK) levels and renal impairment. Despite fluid replacement, neither his blood pressure nor renal function improved, and he was transferred to our hospital.

Upon examination, he showed mild weakness in the extremities, oedema of the extremities, Gottron’s sign over his MCP joints and erythema of his face and upper back. Painful erythematous ulcers were also observed on the skin of his neck, axillae, flexor surfaces of the elbows and popliteal fossae and on the mucosa of his hard palate, tongue and lips.

Blood tests revealed elevated CK levels (5440 U/l), renal dysfunction (creatinine 1.58 mg/dl), haemoconcentration (haematocrit 54.2%) and hypoalbuminemia (albumin 2.2 g/dl). VEGF protein was not elevated. M protein and proteinuria were not detected. He was negative for ANA, but anti-NXP2 antibody was detected by immunoprecipitation followed by western blotting. CT revealed moderate ascites and mild pleural and pericardial effusions, but no evidence of interstitial lung disease. A skin biopsy specimen from the dorsum of the right hand showed a lichenoid tissue reaction, mucin deposition, hyperkeratosis and extravasation of erythrocytes. A muscle biopsy specimen revealed perifascicular atrophy, perivascular lymphocytic infiltration and necrotic fibres, along with increased MHC class I expression by non-necrotic muscle fibres located mainly at the perifascicular region. Although immunostaining for the membrane attack complex on endomysial capillaries was negative, it was possibly due to prior steroid use.

The findings were diagnosed as dermatomyositis complicated by SCLS. At this point, an infectious workup was negative. He required mechanical ventilation on day 3 of hospitalization, followed by venovenous extracorporeal membrane oxygenation (VV-ECMO) and continuous haemodialysis. On day 3, treatment consisting of steroid pulse therapy followed by prednisolone 1.4 mg/kg/day, IVIG, tacrolimus and proton pump inhibitor was initiated. Tacrolimus was selected for its relatively rapid onset of action. On day 12, his CK levels increased to 22 516  U/l but later decreased. His SCLS was also improved, so renal function partially recovered, and ECMO and mechanical ventilation were discontinued by day 20.

On day 23, glucocorticoid therapy was reduced to prednisolone 0.5 mg/kg/day. The following day the patient developed a perforated duodenal ulcer and underwent emergency surgery. Additionally, *Candida albicans* was detected in the ascites, prompting the initiation of antifungal therapy for *Candida* peritonitis. On day 29, the patient developed haemorrhagic shock due to intra-abdominal bleeding. Haemostasis was achieved by transcatheter arterial embolization of a branch of the gastroduodenal artery, but intra-abdominal rebleeding occurred and the patient died on day 37. An autopsy revealed cytomegalovirus-infected cells at the base of the duodenal ulcer.

Our patient with adult-onset anti-NXP2 antibody-positive dermatomyositis presented with severe vasculopathy-related complications, including SCLS and cutaneous and gastrointestinal ulcers. SCLS is thought to manifest as increased vascular permeability due to vascular endothelial damage, which suggests vasculopathy. Dermatomyositis accompanied by SCLS has been reported in paediatric cases and in an anti-TIF1-γ antibody-positive case [3–5], but to our knowledge, no case with anti-NXP2 antibody has been reported in the literature.

The gastrointestinal ulcers of our patient could be attributed to several factors, including the use of high-dose glucocorticoids, malnutrition, cytomegalovirus infection and stress, with vasculopathy also playing a role. Anti-NXP2 antibody-positive cases can present as gastrointestinal ulcers, including fatal cases due to gastrointestinal perforation [6]. In NXP2 antibody-positive dermatomyositis, gastrointestinal lesions are more common in cases with cutaneous ulcers and are considered a poor prognostic indicator [7]. Gastrointestinal perforation is more common in patients with poor response to initial treatment; occurs within 10 months of treatment, with the duodenum being the most common site; and has a reported mortality rate of 38% [8]. Furthermore, our patient had oral ulcers that were assumed to be associated with vasculopathy. Unlike in cases positive for anti-MDA5 antibody, oral ulcers have not, to our knowledge, been documented in anti-NXP2 antibody-positive cases.

In conclusion, the clinical presentation of anti-NXP2 antibody-positive dermatomyositis is diverse and may involve a variety of severe complications associated with vasculopathy. Patients with cutaneous ulcers often have concomitant gastrointestinal ulcers, which can sometimes lead to fatal gastrointestinal perforation. This case, which showed the novel combination of anti-NXP2 antibody-positive dermatomyositis with SCLS and oral ulcers, underscores the complexity of managing this rare autoimmune condition.

## Data Availability

Data are available upon request.
